# Lipodox® (generic doxorubicin hydrochloride liposome injection): in vivo efficacy and bioequivalence versus Caelyx® (doxorubicin hydrochloride liposome injection) in human mammary carcinoma (MX-1) xenograft and syngeneic fibrosarcoma (WEHI 164) mouse models

**DOI:** 10.1186/s12885-017-3377-3

**Published:** 2017-06-06

**Authors:** Vinod Burade, Subhas Bhowmick, Kuntal Maiti, Rishit Zalawadia, Harry Ruan, Rajamannar Thennati

**Affiliations:** 1 Sun Pharma Advanced Research Company Ltd., 17 B Mahal Industrial Estate, Mahakali Caves Road, Andheri (East), Mumbai, Maharashtra 400 093 India; 2 Sun Pharmaceutical Industries Ltd., Sun Pharma Advanced Research Centre (SPARC), Tandalja, Vadodara, Gujarat 390 020 India; 3 Sun Pharma Advanced Research Centre (SPARC), Tandalja, Vadodara, Gujarat 390 020 India

**Keywords:** Anthracycline, Bioequivalence, Doxorubicin HCl liposome injection, In vivo, Preclinical

## Abstract

**Background:**

Doxorubicin (DXR) hydrochloride (HCl) liposome injection is an important part of the treatment armamentarium for a number of cancers. With growing needs for affordable and effective anticancer treatments, the development of generics is becoming increasingly important to facilitate patient access to vital medications. We conducted studies in relevant mouse models of cancer to compare the preclinical antitumour efficacy and plasma pharmacokinetic profile of a proposed generic DXR HCl liposome injection developed by Sun Pharmaceutical Industries Ltd. (SPIL DXR HCl liposome injection) with Caelyx® (reference DXR HCl liposome injection).

**Methods:**

Syngeneic fibrosarcoma (WEHI 164)-bearing BALB/c mice and athymic nude mice transplanted with MX-1 human mammary carcinoma xenografts were treated with SPIL DXR HCl liposome injection, reference DXR HCl liposome injection or placebo, to compare tumour volume, antitumour activity (percentage test/control [%T/C] ratio, tumour regression, and specific tumour growth delay) and toxicity (survival and weight changes) in response to treatment. The pharmacokinetic profile of the SPIL and reference product was also studied in syngeneic fibrosarcoma-bearing mice.

**Results:**

Treatment with either SPIL or reference DXR HCl liposome injection resulted in significant reduction in tumour volume from baseline in both models at all doses tested. High antitumour activity (%T/C ≤ 10) was seen from Day 21 and Day 14 onwards in SPIL and reference DXR HCl liposome injection–treated syngeneic fibrosarcoma-bearing mice, respectively, at 9 mg/kg. Moderate antitumour activity (%T/C ≤ 20) was seen from Day 17 and Day 24 onwards in SPIL and reference DXR HCl liposome injection–treated MX-1-bearing mice, respectively, at 6 mg/kg. No significant differences in tumour volume and %T/C were observed between SPIL and reference DXR HCl liposome injection–treated groups at any dose (*p* ≥ 0.05). Toxicity profiles were considered to be generally comparable. Evaluation of test/reference (A/B) ratios and 90% confidence intervals (CIs) for peak serum concentration (C_max_) and area under the curve (AUC_0-t_, and AUC_0-∞_) demonstrated bioequivalence of SPIL and reference DXR HCl liposome injections.

**Conclusions:**

Establishing similarity is of critical importance during the development of generic treatments. SPIL and reference DXR HCl liposome injections were shown to be comparable with regards to antitumour activity, toxicity and pharmacokinetics.

## Background

Doxorubicin (DXR) hydrochloride (HCl) liposome injection contains the anthracycline DXR in a multicomponent liposomal formulation, in which the pharmaceutical profile of DXR is governed by a complex set of physicochemical, biophysical, and nanotechnological properties [1].

Although DXR has a potent antitumour activity, its use in a clinical setting is limited by its toxicity, of which cardiotoxicity is of special concern [2]. The development of liposomal formulations of DXR is one of several strategies developed to circumvent this limitation [2–4]. Liposomal DXR has reduced cardiotoxicity and improved efficacy compared with free DXR, as demonstrated in preclinical and clinical studies [3, 5–9].

Doxorubicin HCl liposome injection has been shown to offer clinical benefit in AIDS-related Kaposi’s sarcoma (compared with conventional therapy), recurrent ovarian cancer (compared with topotecan), metastatic breast cancer (compared with free DXR), and relapsed or refractory multiple myeloma (in combination with bortezomib over bortezomib monotherapy) [1, 10–13]. It was approved in the United States in 1995 and is currently marketed by Janssen as Doxil® in the United States/Japan and as Caelyx® in the rest of the world. Current licensed product indications include (worldwide) AIDS-related Kaposi’s sarcoma, ovarian carcinoma and multiple myeloma (in combination with bortezomib), and, in the European Union, breast carcinoma in patients at risk of anthracycline cardiotoxicity [10, 14, 15]. Doxorubicin HCl liposome injection is an important part of the treatment armamentarium for the cancers for which it is licensed.

Sun Pharmaceutical Industries Ltd. (SPIL) has developed Lipodox® (SPIL DXR HCl liposome injection) as a generic version of Doxil®/Caelyx®. The main aim of generic medications is to try to help improve access to, and affordability of, essential medications. In February 2013, SPIL DXR HCl liposome injection was approved by the US Food and Drug Administration (FDA). A year earlier, the FDA temporarily allowed the importation of SPIL DXR HCI liposome injection to address a shortage of Doxil® in the United States, because there were no approved generic versions of Doxil® available at the time [15].

Even small differences in the formulation or manufacture of liposomal medical products can alter their distribution characteristics in vivo [16]. Therefore, different liposomal formulations of the same product may have different therapeutic performances, despite achieving similar plasma concentrations [16]. Therefore, the objective of the studies presented in this paper was to compare the preclinical antitumour efficacy and plasma pharmacokinetic (PK) profile of SPIL’s DXR HCl liposome injection with the reference product (Caelyx®) in relevant mouse models of cancer.

Two efficacy studies were conducted, one in syngeneic fibrosarcoma-bearing BALB/c mice and the other in athymic nude mice transplanted with MX-1 human mammary carcinoma xenografts. Plasma pharmacokinetics were evaluated in syngeneic fibrosarcoma-bearing BALB/c mice.

## Methods

### Study treatments

Placebo and SPIL DXR HCl liposome injection (SPIL, Halol, India), and reference DXR HCl liposome injection (Caelyx®, Janssen-Cilag International NV, Beerse, Belgium) were stored at 2–8 °C. SPIL and reference DXR HCl liposome injections contained 2 mg/mL of the active ingredient, DXR HCl. Placebo contained no active ingredient. Placebo matched the SPIL and reference products in all other components, containing N-(carbonyl-methoxypolyethylene glycol 2000)-1,2-distearoyl-sn-glycerol-3-phosphoethanolamine (mPEG-DSPE), hydrogenated soy phosphatidylcholine (HSPC), cholesterol, ammonium sulphate, L-histidine as a buffer, hydrochloric acid and/or sodium hydroxide for pH control, sucrose to maintain isotonicity and water for injection. All products were either used at 2 mg/mL, or diluted to the desired concentration in sterile 5% dextrose solution.

### Cell lines and xenograft tissue

Male BALB/c mice were shaved at the injection site using a shaver and hair-removing cream. Solid tumours were obtained by intradermal inoculation of tumour cells on the shaved portion of dorsal skin. Viable phosphate buffer saline (PBS)–suspended WEHI 164 syngeneic fibrosarcoma cells (efficacy study, 1 × 10^7^ cells per mL; bioequivalence PK study, 2 × 10^7^ cells per mL) were used for tumour induction (cell line [catalogue no. CRL-1751] purchased from American Type Culture Collection [ATCC; Peenya, Bangalore]). Once tumours became palpable, the diameters were measured in three perpendicular planes on alternate days with calibrated digital Vernier callipers. Tumour volume was calculated by using the formula of an ellipse: V = π/6 (D1 × D2 × D3) mm^3^, where D1/D2/D3 is the diameter (mm) in three different planes respectively. Female athymic nude mice were transplanted with MX-1 human mammary carcinoma xenografts (supplied by CLS Cell Lines Service GmbH [Eppelheim, Germany; catalogue no. 300296]) as previously described [17, 18]. Briefly, three donor animals with a tumour diameter ~ 10 mm were euthanised with a high dose of isoflurane (Isorane®) and the tumours were removed. Necrotic material was removed and tumour fragments of ~2 × 2 mm were prepared. Each recipient animal (total 150) was anaesthetised using isoflurane (Isorane®), via the Compact Anaesthesia Workstation (VetTec Solutions; UK), and an individual piece of tumour was inserted into an incision above the base of the tail. Isoflurane is widely used in animal experiments, and was chosen because it is rapidly taken up by, and eliminated from, the body through the lungs. The transplantation procedure was completed within 30 min. These mice were used for experimentation. Tumour volume was calculated using the formula of a sphere: V = [(D1 + D2)/2]^3^ × 0.5236 mm^3^.

### Animals

Animal experiments were conducted according to the guidelines of the Committee for the Purpose of Control and Supervision of Experiments on Animals and was approved by the Institutional Animal Ethics Committee (IAEC/49, meeting no. 6, dated 22.03.04, for syngeneic fibrosarcoma-bearing BALB/c mice study; and IAEC/172, meeting no. 17, dated 19.04.07, for mammary carcinoma xenograft–bearing nude mice and plasma pharmacokinetics study in fibrosarcoma-bearing BALB/c mice) of Sun Pharma Advanced Research Company Ltd. (SPARC).

Male BALB/c mice and female athymic nude mice were supplied by Laboratory Animal Resources (LAR), SPARC. BALB/c mice were used for the syngeneic fibrosarcoma efficacy study and bioequivalence study. Animals used in the efficacy study were 6–8 weeks of age at the time of receipt and weighed 20–25 g. Animals used in the bioequivalence study were 7–10 weeks of age at the time of receipt and weighed 17–28.5 g. For the MX-1 xenograft efficacy study, athymic nude mice 6–10 weeks of age and weighing 20–30 g were received. For BALB/c and athymic nude mice, a veterinary health check was performed before tumour induction to select healthy animals.

Male BALB/c mice and female athymic nude mice used in the studies were housed in individually ventilated polysulfone cages. Male BALB/c mice used in the efficacy study were housed individually, while those used in the PK bioequivalence study were housed four per cage prior to tumour induction and individually thereafter. Female athymic nude mice were housed individually after tumour induction. Cages were maintained under constant temperature (18–26 °C), humidity (30%–70%) and lighting conditions (12 h light and 12 h dark). Animals received reverse osmosis (RO) water supplied by LAR, and Harlan Teklad Rodent Diet 2018 (BALB/c mice) or Harlan Rodent Diet 2019 IR (athymic nude) *ad libitum*.

### Study design

The respective designs of the efficacy and bioequivalence studies are shown in Table 1. Fibrosarcoma-bearing male BALB/c mice were used in the syngeneic efficacy study (Table 1a). Animals were screened and selected for randomisation into treatment groups on the basis of body weight and tumour volume, weighed 20–25 g (mean [± SEM] 24.49 g [0.33]), and had a tumour volume ~ 60–120 mm^3^ at randomisation. The aim was to have a minimum of 10 animals per group to get meaningful conclusions after statistical evaluation. The animals were randomly assigned to different treatment groups (*n* = 11 to *n* = 15). Intravenous injections of SPIL or reference DXR HCl liposome injection (9.0 mg/kg), or placebo were administered on Days 0, 7, 14 and 21. The animals were treated and assessed in the order of their study identification numbers allocated at randomisation. This meant that animals in the placebo group were treated first, followed by the test group and then the reference group. Tumour diameters and body weight were recorded weekly over the 28-day study period. For humane reasons, animals were euthanised when tumour volume reached >4000 mm^3^. The animals were checked throughout the study for deaths. All surviving animals were euthanised by CO_2_ on the last day of the study.

**Table 1:** Study design

Group no.	Dose groups	Doses (mg/kg)		Concentrations (mg/mL)	Dose volume (mL/kg)	No. of animals
a. Treatment groups for the syngeneic fibrosarcoma tumour efficacy study						
1	Placebo	Nil		Nil	4.5	15
2	SPIL DXR HCl liposome injection	9.0		2.0	4.5	11
3	Reference DXR HCl liposome injection	9.0		2.0	4.5	15
Total number						41
b. Treatment groups for the human mammary carcinoma xenograft efficacy study						
1	Placebo	Nil		Nil	10.0	10
2	SPIL DXR HCl liposome injection	1.5		0.6	2.5	10
3		3.0		0.6	5.0	10
4		6.0		0.6	10.0	10
5	Reference DXR HCl liposome injection	1.5		0.6	2.5	10
6		3.0		0.6	5.0	10
7		6.0		0.6	10.0	10
Total number						70
Group no.	Dose groups	Doses (mg/kg)	Concentrations (mg/mL)	Dose volume (mL/kg)	Time point (hour)	No. of animals per time point
c. Study groups for the syngeneic fibrosarcoma tumour bioequivalence study						
1	SPIL DXR HCl liposome injection	5	1	5	0.08	12
					0.5	12
					3	12
					5	12
					8	12
					24	12
					48	12
					96	12
					168	12
					240	12
2	Reference DXR HCl liposome injection	5	1	5	0.08	12
					0.5	12
					3	12
					5	12
					8	12
					24	12
					48	12
					96	12
					168	12
					240	12
Total number						240

*Abbreviations*: *DXR* doxorubicin, *HCl* hydrochloride, *SPIL* Sun Pharmaceutical Industries Ltd.

MX-1-bearing female athymic nude mice were used in the xenograft efficacy study (Table 1b). Animals were screened and selected for randomisation into treatment groups on the basis of body weight and tumour volume, weighed 20–30 g (mean [± SEM] 22.21 g [0.17]), and had a tumour volume ~ 65–180 mm^3^ at randomisation. As above, the order of treatment and assessment was determined by the animal’s identification number. Intravenous injections of SPIL or reference DXR HCl liposome injection (1.5 mg/kg, 3.0 mg/kg or 6.0 mg/kg), or placebo were administered on Days 0, 7 and 14. Tumour diameter and body weights were recorded twice weekly over the 42-day study period. For humane reasons, animals were euthanised when tumour volume reached >4000 mm^3^. The animals were checked daily throughout the study for deaths. Syngeneic fibrosarcoma-bearing BALB/c mice used in the PK bioequivalence analysis were screened and selected for randomisation into study groups on the basis of tumour volume (Table 1c). Animals weighed between 17 and 28.5 g (mean [± SEM] 24.49 g [0.33]) and had a mean tumour volume of 90 ± 30 mm^3^ per group (time point) at randomisation. Intravenous injections were administered on Day 0. Samples were collected at 0.08, 0.5, 3, 5, 8, 24, 48, 96, 168 and 240 h post injection and animals were euthanised at this time. All groups were treated on the same day; the test group was treated/assessed before the reference group.

### Experimental outcomes

Percentage test/control (%T/C) was calculated as follows: (mean tumour volume of drug treated group on Day X) / (mean tumour volume of control group on Day X) × 100, where X was the day of observation. According to the US National Cancer Institute (NCI), Division of Cancer Treatment criteria, %T/C ≤ 42 is considered to indicate minimum acceptable antitumour activity (biological relevance), %T/C ≤ 20 is considered to indicate moderate antitumour activity, and %T/C ≤ 10 is considered to indicate a high antitumour activity. The optimal %T/C value for each group was the minimal %T/C ratio, thus reflecting the maximal tumour growth inhibition. Tumour regression was recorded as partial (PR) if the tumour volume decreased to less than 50% of the tumour volume at the start of the treatment without dropping below measurable size, or as complete (CR) if the tumour burden had become impalpable. Specific tumour growth delay (SGD) is defined as the difference in median time for drug-treated tumours and placebo (control)–treated tumours to reach a given volume (V) divided by the median time for the control tumours to reach the same volume (V), where V is tumour volume after two volume doublings from initial tumour volume at the start of treatment. However, if V was not achieved in the test or reference group animal until the last day of the study, the same value was considered as time to reach the given volume for that animal. The efficacy criterion for SGD was >1. Mouse body weight changes were calculated as (mouse weight on Day X – mouse weight on Day 0) / (mouse weight on Day 0) × 100, where X was the day of observation. A dose producing a mean weight loss ≥15% of initial body weight was considered toxic [19, 20]. Recovery of weight after completion of treatment was also evaluated. Survival analysis was done by Kaplan-Meier method. Plasma concentration of DXR was determined by liquid chromatography–tandem mass spectrometry (LC-MS/MS). Plasma concentration data were used in the calculation of peak serum concentration (C_max_); area under the curve (AUC_0-t_ and AUC_0-∞_); time taken to reach the maximum concentration (T_max_); elimination of half-life (T_1/2_); elimination rate constant (K_el_); and confidence intervals (CI).

### Statistical analysis

Tumour volume and body weight values are presented as the mean ± standard error of mean (SEM) and percent change, respectively. Tumour volume and %T/C data were analysed using two-way analysis of variance (ANOVA) followed by Bonferroni post tests, and body weight data were analysed using one-way ANOVA followed by Dunnett’s *t*-test. The Kaplan-Meier method was used to estimate survival, and differences were analysed by log-rank test. Statistical analyses were carried out with GraphPad Prism, and *p* values <0.05 were considered significant. Plasma concentration data were used in the calculation of PK parameters by noncompartmental intravenous-bolus input model (WinNonlin 5.0). Geometric least-squares mean values are represented for Ln-transformed C_max_, AUC_0-t_ and AUC_0-∞_. Ln-transformed data were analysed using ANOVA.

## Results

### Antitumour efficacy

**Fig. 1:**
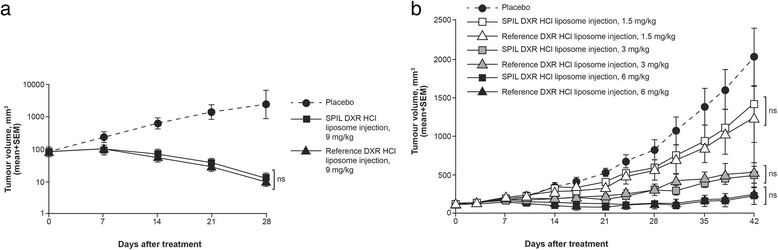
Change in tumour volumes following treatment with DXR HCl liposome injections or placebo. The figure shows: **a** change in tumour volumes in syngeneic fibrosarcoma-bearing mice treated with the SPIL DXR HCl liposome injection (*n* = 11), reference DXR HCl liposome injection (*n* = 15) or placebo (*n* = 15). One animal in the reference DXR HCl liposome group was found dead on Day 28. **b** Change in tumour volumes in MX-1-bearing mice treated with either the SPIL DXR HCl liposome injection, reference DXR HCl liposome injection or placebo (*n* = 10 per group). Two animals in the 6 mg/kg SPIL DXR HCl liposome injection group were found dead: one on Day 28 and one on Day 35. Two animals in the reference DXR HCl liposome group were found dead: one on Day 28 and one on Day 38. In both studies, differences were analysed by two-way analysis of variance followed by Bonferroni post tests. *P* values <0.05 were considered significant. Error bars indicate the standard error of mean for replicate animals. Abbreviations: DXR, doxorubicin; HCl, hydrochloride; ns, non-significant; SEM, standard error of mean; SPIL, Sun Pharmaceutical Industries Ltd.

Mean tumour volume data for syngeneic fibrosarcoma-bearing and MX-1-bearing mice, treated with either SPIL DXR HCl liposome injection, reference DXR HCl liposome injection, or placebo, are shown in Fig. 1. In syngeneic fibrosarcoma-bearing mice, a significant reduction in tumour volume was seen from Day 14 onwards in both 9 mg/kg DXR HCl liposome injection–treated groups (*p* < 0.01 vs placebo; Fig. 1a). No significant difference in mean tumour volume was observed between the SPIL and reference DXR HCl liposome injection–treated groups. In MX-1-bearing mice, a significant reduction in tumour volume was seen from Day 35 onwards in both 1.5 mg/kg DXR HCl liposome injection–treated groups (SPIL product, *p* < 0.05 vs placebo; reference product *p* < 0.01 vs placebo; Fig. 1b); from Day 24 and 28 onwards in the 3 mg/kg SPIL and reference DXR HCl liposome injection–treated groups, respectively (both *p* < 0.05 vs placebo; Fig. 1b); and from Day 21 and 24 onwards in the 6 mg/kg SPIL and reference DXR HCl liposome injection–treated groups, respectively (SPIL product, *p* < 0.05 vs placebo; reference product *p* < 0.01 vs placebo; Fig. 1b). No significant difference in mean tumour volume was observed between the SPIL and reference DXR HCl liposome injection-treated groups at any dose.

Antitumour activities of SPIL and reference DXR HCl liposome injections were assessed and compared based on %T/C (Table 2). In syngeneic fibrosarcoma-bearing mice, a high antitumour activity (%T/C ≤ 10, based on NCI criteria) was seen from Day 21 and Day 14 onwards in the SPIL and reference DXR HCl liposome injection–treated groups, respectively, at a dose of 9 mg/kg (optimal %T/C values were 0.48 and 0.4 for SPIL and reference products, respectively). Acceptable antitumour activity (%T/C ≤ 42, based on NCI criteria) was seen from Day 7 onwards in both DXR HCl liposome injection–treated groups. No significant difference in %T/C was observed between the SPIL and reference DXR HCl liposome injection–treated groups. In MX-1-bearing mice, no biologically significant antitumour activity was seen in the SPIL and reference DXR HCl liposome injection–treated groups as measured by %T/C, at a dose of 1.5 mg/kg (optimal %T/C values were 67.03 and 61.12, both at Day 35); acceptable antitumour activity (%T/C ≤ 42, based on NCI criteria) was seen after Day 21 in both DXR HCl liposome injection–treated groups, at a dose of 3 mg/kg (optimal %T/C values were 24.61 and 25.86, both at Day 42); and moderate antitumour activity (%T/C ≤ 20, based on NCI criteria) was seen from Day 17 and Day 24 onwards in the SPIL and reference DXR HCl liposome injection–treated groups, respectively, at a dose of 6 mg/kg (optimal %T/C values were 10.44 and 11.65, at Days 38 and 42, for the SPIL and reference products, respectively). No significant difference in %T/C was observed between the SPIL and reference DXR HCl liposome injection–treated groups at any dose.

**Table 2:** Comparison of %T/C data of SPIL and reference DXR HCl liposome injections^a^

	Syngeneic fibrosarcoma mouse model		Human mammary carcinoma xenograft mouse model					
Days	SPIL DXR HCl liposome injection 9 mg/kg	Reference DXR HCl liposome injection 9 mg/kg	SPIL DXR HCl liposome injection 1.5 mg/kg	Reference DXR HCl liposome injection 1.5 mg/kg	SPIL DXR HCl liposome injection 3 mg/kg	Reference DXR HCl liposome injection 3 mg/kg	SPIL DXR HCl liposome injection 6 mg/kg	Reference DXR HCl liposome injection 6 mg/kg
	%T/C (n)	%T/C (n)	%T/C (n)	%T/C (n)	%T/C (n)	%T/C (n)	%T/C (n)	%T/C (n)
0	98.26 (11)	96.59 (15) ns	109.73 (10)	104.01 (10) ns	121.01 (10)	110.15 (10) ns	106.77 (10)	119.41 (10) ns
3			95.85 (10)	99.43 (10) ns	105.52 (10)	100.33 (10) ns	90.24 (10)	105.11 (10) ns
7	41.83 (11)	41.81 (15) ns	99.89 (10)	96.20 (10) ns	92.40 (10)	84.05 (10) ns	73.89 (10)	86.59 (10) ns
10			101.32 (10)	113.86 (10) ns	80.23 (10)	90.67 (10) ns	57.70 (10)	67.29 (10) ns
14	11.07 (11)	8.46 (15) ns	96.73 (10)	82.17 (10) ns	56.76 (10)	56.82 (10) ns	27.63 (10)	39.29 (10) ns
17			82.60 (10)	70.85 (10) ns	50.36 (10)	50.02 (10) ns	18.47 (10)	30.75 (10) ns
21	2.56 (11)	2.00 (15) ns	77.17 (10)	61.13 (10) ns	33.51 (10)	42.06 (10) ns	13.80 (10)	20.37 (10) ns
24			77.12 (10)	69.97 (10) ns	33.53 (10)	36.99 (10) ns	14.29 (10)	16.82 (10) ns
28	0.48 (11)	0.40 (14) ns	71.72 (10)	67.15 (10) ns	35.25 (10)	37.60 (10) ns	12.90 (9)	13.92 (9) ns
31			70.05 (10)	64.51 (10) ns	27.48 (10)	39.35 (10) ns	10.64 (9)	11.29 (9) ns
35			67.03 (10)	61.12 (10) ns	28.80 (10)	31.64 (10) ns	10.65 (8)	11.19 (9) ns
38			68.52 (10)	63.41 (10) ns	28.22 (10)	31.29 (10) ns	10.44 (8)	11.75 (8) ns
42			70.10 (10)	61.24 (10) ns	24.61 (10)	25.86 (10) ns	10.73 (8)	11.65 (8) ns

*Abbreviations*: *DXR* doxorubicin, *HCl* hydrochloride, *ns* non-significant as compared to SPIL DXR HCl liposome injection, *SPIL* Sun Pharmaceutical Industries Ltd., *%T/C* percentage test/control

^a^Percentage test/control (%T/C) data were calculated from tumour volume data. %T/C ≤ 42% indicates acceptable antitumour activity; %T/C ≤ 20% indicates moderate antitumour activity; %T/C ≤ 10% indicates high antitumour activity. Differences were analysed by two-way analysis of variance followed by Bonferroni post tests. *P* values <0.05 were considered significant.

The antitumour activities of SPIL and reference DXR HCl liposome injections were also assessed and compared based on SGD and other parameters (Table 3). In syngeneic fibrosarcoma-bearing mice, the median time for placebo-treated tumours to reach two tumour volume doublings from initial tumour volume at randomisation was 14 days. Both DXR HCl liposome injection–treated groups showed an SGD value >1, at a dose of 9 mg/kg, which indicates a significant delay in tumour growth compared with the placebo group. Partial tumour regression was observed in 8/11 and 7/15 animals, respectively, for the SPIL and reference DXR HCl liposome injection–treated groups. Complete tumour regression was observed in 3/11 and 7/15 animals, respectively, for the SPIL and reference DXR HCl liposome injection–treated groups; the median duration of complete tumour regression in days (DCR) was 21 and 28 days, respectively, and none of the animals showed tumour recurrence 1 week after last treatment. In MX-1-bearing mice, the median time for placebo-treated tumours to reach two tumour volume doublings was 19 days. Both 6 mg/kg DXR HCl liposome injection–treated groups showed an SGD value >1, which indicates a significant delay in tumour growth compared with the placebo group. At a dose of 6 mg/kg, the SGD was comparable between DXR HCl liposome injection–treated groups. Partial tumour regression was observed in 2/10 animals in each of the 6 mg/kg SPIL and reference DXR HCl liposome injection–treated groups. Complete tumour regression was observed in 1/10 animals for each of the 3 mg/kg and 6 mg/kg SPIL DXR HCl liposome injection–treated groups, but not for any animals in any of the other groups. Of the two animals with complete regression, the median DCR was 38 and 28 days, respectively, for 3 mg/kg and 6 mg/kg SPIL DXR HCl liposomal injection, and none of the animals showed tumour recurrence.

**Table 3:** Comparison of SGD and other parameters of SPIL and reference DXR HCl liposome injections^a^

Treatment (dose)	Median tumour volume (days)	SGD	CR/total (n/n)	PR/total (n/n)	DCR (days)	TFS/TR (n/n)
Syngeneic fibrosarcoma mouse model						
Placebo	14	-	0/15	-	-	0/0
SPIL DXR HCl liposome injection 9 mg/kg	>28^b^	>1	3/11	8/11	21	3/0
Reference DXR HCl liposome injection 9 mg/kg	>28^b^	>1	7/15	7/15	28	7/0
Human mammary carcinoma xenograft mouse model						
Placebo	19.00	-	-	-	-	-
SPIL DXR HCl liposome injection 1.5 mg/kg	28.00	0.5	0/10	0/10	NA	NA
Reference DXR HCl liposome injection 1.5 mg/kg	28.00	0.5	0/10	0/10	NA	NA
SPIL DXR HCl liposome injection 3 mg/kg	35.00	0.8	1/10	0/10	38	1/0
Reference DXR HCl liposome injection 3 mg/kg	28.00	0.5	0/10	0/10	NA	NA
SPIL DXR HCl liposome injection 6 mg/kg	42.00	1.2	1/10	2/10	28	1/0
Reference DXR HCl liposome injection 6 mg/kg	>42^c^	>1.2	0/10	2/10	NA	NA

*Abbreviations*: *CR/total (n/n)* animals with complete tumour regression/total no. of animals, *DCR* median duration of complete tumour regression in days, *DXR* doxorubicin, *HCl* hydrochloride, *PR/total (n/n)* animals with partial tumour regression/total no. of animals, *SGD* specific tumour growth delay, *SPIL* Sun Pharmaceutical Industries Ltd., *TFS/TR* no. of tumour free survivors/no. of tumour recurrences

^a^SGD and other parameters were calculated from tumour volume data. The efficacy criteria for SGD parameter was >1. Tumour regression was recorded as partial (PR) if the tumour volume decreased to less than 50% of the tumour volume at the start of the treatment without dropping below measurable size, or as complete (CR) if the tumour burden had become impalpable.

^b^None of the animals reached two volume doublings from initial tumour volume at the start of treatment. Hence median time to reach two tumour volume doublings was considered as >28 days.

^c^None of the animals reached two volume doublings from initial tumour volume at the start of treatment. Hence median time to reach two tumour volume doublings was considered as >42 days.

Based on these results, the antitumour activities of SPIL and reference DXR HCl liposome injections were considered to be comparable at all doses tested in both models.

### Safety

**Fig. 2:**
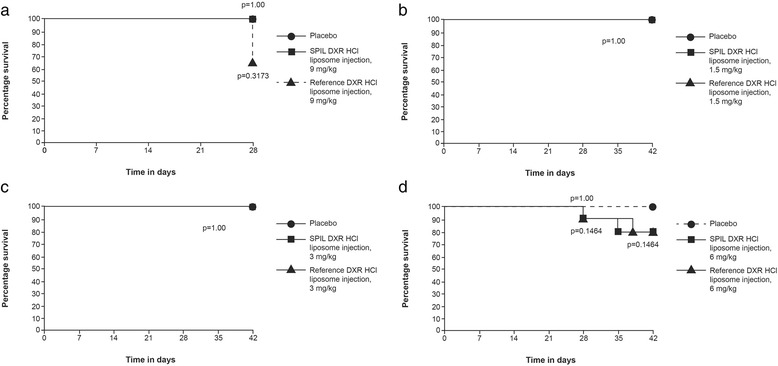
Kaplan-Meier plots for fibrosarcoma- and mammary tumour-bearing mice receiving DXR HCl liposome injections or placebo. The figure shows data for: **a** syngeneic fibrosarcoma-bearing mice receiving 9 mg/kg SPIL DXR HCl liposome injection (*n* = 11), reference DXR HCl liposome injection (*n* = 15) or placebo (*n* = 15); **b** MX-1-bearing mice receiving 1.5 mg/kg SPIL or reference DXR HCl liposome injection, or placebo (*n* = 10 in each group); **c** MX-1-bearing mice receiving 3 mg/kg SPIL or reference DXR HCl liposome injection, or placebo (*n* = 10 in each group); and **d** MX-1-bearing mice receiving 6 mg/kg SPIL or reference DXR HCl liposome injection, or placebo (*n* = 10 in each group). Survival was estimated using the Kaplan-Meier method, and differences were analysed by log-rank test. Abbreviations: DXR, doxorubicin; HCl, hydrochloride; SPIL, Sun Pharmaceutical Industries Ltd.

The toxicity profiles of SPIL and reference DXR HCl liposome injections were assessed and compared based on percentage survival data and changes in body weight (Fig. 2).

In syngeneic fibrosarcoma-bearing mice, one animal in the reference DXR HCl liposome group was found dead on Day 28. No significant difference in survival proportions was observed between either DXR HCl liposome injection–treated group and placebo, or between the SPIL and reference DXR HCl liposome injection–treated groups (*p* > 0.05; Fig. 2a). In MX-1-bearing mice, two animals in the 6 mg/kg SPIL DXR HCl liposome injection group were found dead (one on Day 28 and one on Day 35) and two animals in the 6 mg/kg reference DXR HCl liposome group were found dead (one on Day 28 and one on Day 38). No significant difference in survival proportions was observed between either DXR HCl liposome injection–treated group and placebo, or between SPIL and reference DXR HCl liposome injection–treated groups at any dose (*p* > 0.05; Fig. 2b–d).

**Fig. 3:**
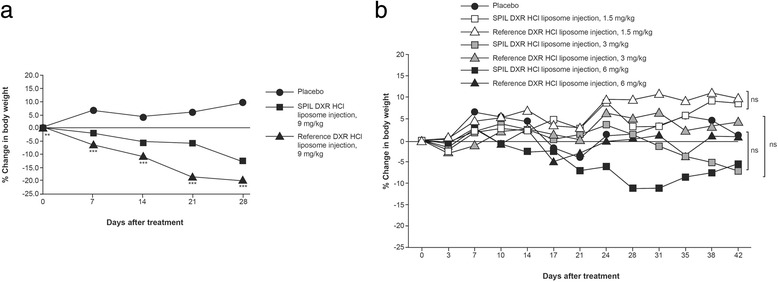
Changes in body weight following treatment with DXR HCl liposome injections or placebo. The figure shows the change in body weight for: **a** syngeneic fibrosarcoma-bearing mice receiving SPIL DXR HCl liposome injection (*n* = 11), reference DXR HCl liposome injection (*n* = 15) or placebo (*n* = 15). One animal in the reference DXR HCl liposome group was found dead on Day 28; and **b** MX-1-bearing mice receiving SPIL DXR HCl liposome injection (1.5, 3 or 6 mg/kg); reference DXR HCl liposome injection (1.5, 3 or 6 mg/kg) or placebo (*n* = 10 per treatment group). Two animals in the 6 mg/kg SPIL DXR HCl liposome injection group were found dead, one on Day 28 and one on Day 35; two animals in the 6 mg/kg reference DXR HCl liposome group were found dead, one on Day 28 and one on Day 38. A dose producing a weight loss ≥15% of initial body weight was considered toxic. Body weight data were analysed by one-way analysis of variance followed by Dunnett’s *t*-test. **p < 0.01; ***p < 0.001; ns, non-significant compared with the SPIL DXR HCl liposome injection. Abbreviations: DXR, doxorubicin; HCl, hydrochloride; ns, non-significant; SPIL, Sun Pharmaceutical Industries Ltd.

In syngeneic fibrosarcoma-bearing mice, significant decreases in body weight compared with baseline were seen from Day 28 in the SPIL DXR HCl liposome injection–treated group, and from Day 7 in the reference DXR HCl liposome injection–treated group (Fig. 3a). A significant increase in body weight was observed from Day 28 in the placebo-treated group. The maximum decrease in body weight observed in the SPIL and reference DXR HCl liposome injection groups was 12.6% and 19.9%, respectively, at a dose of 9 mg/kg.

In MX-1-bearing mice, no significant decreases in body weight compared with baseline were observed at any dose in any of the treatment groups, when compared with their respective Day 0 weight, except on Day 31 and Day 38 for the reference DXR HCl liposome injection–treated group, at a dose of 1.5 mg/kg (Fig. 3b). The maximum decrease in body weight observed in the SPIL and reference DXR HCl liposome injection groups was 11.3% and 5%, respectively, at the highest dose tested. No significant difference in body weight was observed between the SPIL and reference DXR HCl liposome injection–treated groups at any dose.

Based on these results, the toxicity profile of SPIL DXR HCl liposome injection was considered to be generally comparable to reference DXR HCl liposome injection.

### Pharmacokinetics

The geometric mean peak DXR plasma concentrations (C_max_) were approximately 120.91 and 125.64 μg/mL following injection of 5 mg/kg SPIL DXR HCl liposome and reference DXR HCl liposome, respectively (Table 4). The AUC_0-t_ and AUC_0-∞_ values were 3002.27 and 3355.09 μg·hr/mL, respectively, for SPIL DXR HCl liposome injection, and 3166.07 and 3444.65 μg·hr/mL, respectively, for reference DXR HCl liposome injection. The C_max_, AUC_0-t_ and AUC_0-∞_ values were comparable between SPIL and reference DXR HCl liposome injections. There were no significant differences between the products with respect to T_max_, T_1/2_, and K_el_. The A/B ratios (reference as B:SPIL as A) and 90% confidence intervals (CIs) for C_max_, AUC_0-t_ and AUC_0-∞_ for SPIL and reference DXR HCl liposome injections were within the 80%–125% range, indicating that the two products are bioequivalent in fibrosarcoma-bearing mice.

**Table 4:** Summary statistics based on pharmacokinetic data from DXR concentrations in plasma of fibrosarcoma-bearing mice^a^

Parameter	Ln AUC_0-t_ (μg·hr./ml)^b^	Ln AUC_0-∞_ (μg·hr./ml)^b^	Ln C_max_ (μg/mL)^b^	T_max_ (hr)	T_1/2_ (hr)	K_el_ (hr^−1^)
SPIL DXR HCl liposome injection (batch no. LD-72)						
Geometric mean	3002.27	3355.09	120.91	0.13	22.72	0.03
CV%	11.3	13.7	8.2	102.7	21.7	20.1
Reference DXR HCl liposome injection (lot no. 070382820)						
Geometric mean	3166.07	3444.65	125.64	0.17	27.40	0.03
CV%	11.3	10.9	7.8	84.8	22.7	20.4
Least-squares means ratio (reference as B:SPIL as A)						
A/B ratio	94.83	97.40	96.24			
90% confidence interval	87.64–102.60	89.31–106.23	91.12–101.65			
Analysis of variance						
Form	0.2593	0.6071	0.2411			
Power	0.9977	0.9935	1.0000			
Interanimal CV%	11.27	12.42	7.81			

*Abbreviations*: *AUC* area under the curve, *C*_*max*_ peak serum concentration, *CV%* coefficient of variation, *DXR* doxorubicin, *HCl* hydrochloride, *K*_*el*_ elimination rate constant, *SPIL* Sun Pharmaceutical Industries Ltd., *T*_*1/2*_ elimination of half-life, *T*_*max*_ time taken to reach the maximum concentration

^a^The pharmacokinetic profiles of SPIL and reference DXR HCl liposome injections were evaluated following single intravenous injection in syngeneic fibrosarcoma (WEHI 164)-bearing BALB/c mice. Blood samples were collected at 0.08, 0.50, 3.00, 5.00, 8.00, 24.00, 48.00, 96.00, 168.00 and 240.00 h post injection. Twelve animals were used for a single time point per group. Plasma concentration data were used in the calculation of pharmacokinetic parameters by noncompartmental intravenous-bolus input model (WinNonlin 5.0).

^b^Geometric least-squares mean values are represented.

## Discussion

GenRajamannar.Thennati@sunpharma.comeric liposomal medications are complex and must be shown to have an equivalent efficacy and safety to the originator. In general, head-to-head analytical and nonclinical similarity assessments against the originator are required before they are tested in human studies [16]. Sun Pharmaceuticals Industries Ltd.’s DXR HCl liposome injection has been assessed in several in vivo preclinical studies, three of which are presented in this paper (data on file). It has also been assessed in four clinical trials in patients with breast cancer, ovarian cancer and multiple myeloma (data on file).

The objective of the studies presented in this paper was to compare the preclinical antitumour efficacy, safety and plasma PK profile of SPIL’s DXR HCl liposome injection with reference DXR HCl liposome injection in relevant mouse models. The MX-1 xenograft model was used because it is a well-established model for human breast cancer, one of the indications of the reference product. A syngeneic fibrosarcoma model was also used to study how SPIL DXR HCl liposome injection and the reference product compare in the presence of a functional immune system.

Treatment with either SPIL or reference DXR HCl liposome injection resulted in a significant reduction in tumour volume in both animal models and at all doses tested, with no significant differences observed between SPIL and reference DXR HCl liposome injection–treated groups. With regards to antitumour activity, SPIL and reference DXR HCl liposome injections were considered to be comparable. The toxicity profile of SPIL DXR HCl liposome injection was generally comparable to reference DXR HCl liposome injection in fibrosarcoma-bearing BALB/c mice. Weekly dosing of the reference product at 9 mg/kg produced a weight loss ≥15% of initial body weight in BALB/c mice, which was considered toxic. However, we acknowledge that the baseline body weight data were statistically significantly different between groups, which may have affected this particular result. At all three doses, the safety profile (based on mortality and body weight) of SPIL DXR HCl liposome injection was comparable to reference DXR HCl liposome injection in MX-1-bearing athymic nude mice following weekly dosing. The rate and extent of DXR absorption in fibrosarcoma-bearing mice were similar between the SPIL and reference DXR HCl liposome injections. The A/B ratios and 90% CIs for C_max_, AUC_0-t_ and AUC_0-∞_ for SPIL and reference DXR HCl liposome injections were within the 80%–125% range, indicating that the two products are bioequivalent in fibrosarcoma-bearing mice.

Collectively, these results demonstrate that SPIL and reference DXR HCl liposome injections have comparable PK and pharmacodynamic profiles. They also demonstrate that similar plasma DXR concentrations between these two products correlate with similar therapeutic performance.

The present studies have been conducted in line with guidance proposed by the European Medicines Agency (EMA) [21] and form part of a program of studies designed to demonstrate similarity between the SPIL DXR HCl liposome injection and Caelyx® and Doxil®. The program included other preclinical studies in rodent models of breast cancer and ovarian cancer that assessed comparative plasma and tissue distribution, toxicity, and in vitro haemolytic potentiality; clinical studies in patients with breast or ovarian cancer or multiple myeloma to assess bioequivalence and safety; and physicochemical equivalence studies (structure, content and stability of liposomes in vitro and in vivo). These studies have now been completed, and it is anticipated that they will be published during 2017.

The availability of a generic-version DXR HCl liposome injection could potentially improve access to and affordability of an established and important cancer therapy. SPIL’s DXR HCI liposome injection is currently being marketed in a number of countries, including the United States. It has also been used to address a shortage of Caelyx® in several European countries, under a special importation program (data on file). Further research is required to characterize the positive impact that SPIL DXR HCl liposome injection might have on patients with cancer. However, the results of the present studies support the further development of SPIL DXR HCl liposome injection.

## Conclusion

The primary objective of these preclinical studies was to compare the antitumour efficacy and safety of SPIL DXR HCl liposome injection with reference DXR HCl liposome injection in syngeneic fibrosarcoma-bearing BALB/c mice and MX-1-bearing athymic nude mice. Another objective was to compare the PK profile of SPIL DXR HCl liposome injection with reference DXR HCl liposome injection in fibrosarcoma-bearing BALB/c mice. These studies have demonstrated that the efficacy and toxicity profile of SPIL DXR HCl liposome injection was comparable to reference DXR HCl liposome injection in both efficacy studies and at all doses tested. Furthermore, the rate and extent of DXR absorption in mice were similar between SPIL DXR HCl liposome injection and reference DXR HCl liposome injection in fibrosarcoma-bearing BALB/c mice.

## Abbreviations

ANOVA: Analysis of variance
AUC: Area under the curve
CI: Confidence interval
CPCSEA: Committee for the Purpose of Control and Supervision of Experiment on Animals
CR: Complete regression
DCR: Median duration of complete tumour regression in days
DXR: Doxorubicin
EMA: European Medicines Agency
FDA: US Food and Drug Administration
HCl: Hydrochloride
HSPC: Hydrogenated soy phosphatidylcholine
IAEC: Institutional Animal Ethics Committee
LAR: Laboratory Animal Resources
LC-MS/MS: Liquid chromatography–tandem mass spectrometry
NCI: US National Cancer Institute
PBS: Phosphate buffered saline
PK: Pharmacokinetic
PR: Partial regression
SEM: Standard error of the mean
SGD: Specific tumour growth delay
SPARC: Sun Pharma Advanced Research Centre
SPIL: Sun Pharmaceutical Industries Ltd.
T/C: Test/control
V: Volume
